# The Synergistic Utilization of Glass Aggregates and Glass Powder on the Thermal and Mechanical Properties of Concrete

**DOI:** 10.3390/ma18102405

**Published:** 2025-05-21

**Authors:** Bo Wen, Huaizheng Wang, Guanyi Gao, Lu Zhang, Zhengyao Yu, Zhihao Wang

**Affiliations:** 1 School of Civil Engineering, Xi’an University of Architecture and Technology, Xi’an 710055, China; wenbo@xauat.edu.cn (B.W.);; 2 Key Laboratory of Structural Engineering and Seismic Education, Xi’an University of Architecture and Technology, Xi’an 710055, China; 3 State Key Laboratory of Green Building, Xi’an University of Architecture and Technology, Xi’an 710055, China; 4 School of Materials Science and Engineering, Xi’an University of Architecture and Technology, Xi’an 710055, China; 5 Xuzhou Survey and Design Branch, State Grid Jiangsu Electric Power Design Consulting Co., Ltd., Xuzhou 221000, China

**Keywords:** composite waste glass concrete, high temperature, thermal properties, replacement method, mechanical properties

## Abstract

Enhancing the utilization rate of waste glass in concrete is crucial for achieving solid waste reduction and low carbon emissions in the construction industry. This study employs the method of simultaneously replacing fine aggregate and cementitious materials in concrete with glass sand and glass powder to prepare composite waste glass concrete (CGC). The compressive strength, alkali–silicate expansion, and thermal properties of CGC were investigated experimentally. The experimental results show that the pozzolanic activity of fine glass powder in CGC can effectively mitigate the ASR reaction, enhance glass utilization, and allow the glass content to reach up to 17.79% of the total concrete mass. The thermal conductivity of the compounded waste glass concrete decreased linearly with increasing temperature, and the specific heat capacity showed three distinct peaks in the range of 180–800 °C, which were caused by chemical dehydration, quartz phase transition, and CaCO_3_ decarbonization, respectively. Furthermore, to examine the impact of replacement mode on the high-temperature resistance of waste glass concrete, the residual strength, physical properties, and microstructure of the concrete were evaluated. It was found that the residual strength ratio of CGC (0.73) exhibited a distinct advantage at 600 °C. At this time, the melting effect of glass can reduce the pore size of concrete and transform large pores into capillary pores. However, as the temperature rises to 800 °C, the melting effect of glass no longer alleviates the high-temperature damage to concrete, and the degree of decomposition of hydration products determines the concrete strength.

## 1. Introduction

Glass has excess production capacity and high waste and is not easily degradable, making it one of the most challenging urban solid wastes to manage worldwide. However, glass exhibits excellent chemical stability and low permeability, making it a suitable filler to replace natural aggregates in concrete [1]. Moreover, the pozzolanic activity of glass powder can also serve as a supplementary cementitious material [2]. After crushing, glass demonstrates physical and mechanical properties similar to natural aggregate. It was first used in concrete in the 1960s to alleviate problems such as over-exploitation of natural sand and gravel brought about by the rapid development of the construction industry [3]. By reducing the demand for natural aggregates in building materials, the construction industry can move towards solid waste utilization and low-carbon development [4].

Glass can not only replace natural sand and gravel in concrete, promoting the recycling of waste materials, but also improve the thermal insulation properties of concrete [5,6]. Therefore, in order to maximize the advantages of waste glass, scholars first applied it to concrete coarse aggregate. However, the limitations of the glass production process (micro-cracks are prone to occur during glass crushing) lead to significant stress concentrations in concrete [7,8]. Therefore, some scholars reduce the glass particle size to replace the concrete fine aggregate. Yan et al. [9] noted that the molten and re-solidifying properties of glass particles heal high-temperature cracks in concrete, mitigating the rate of strength loss after exposure to elevated temperatures, with the greatest post-heating strength reduction occurring between 300 °C and 450 °C [10]. However, it has been found that glass contains a significant amount of amorphous silicon dioxide, which reacts with the alkalis in cement, leading to an alkali–silica reaction (ASR) [11]. Subsequent scholars have revealed that the expansion rate is mainly affected by the glass particle size [12,13,14]. Glass exhibits pozzolanic activity when its particle size is less than 300 μm, reducing the available alkali content in concrete and effectively mitigating concrete expansion. Therefore, some researchers [15,16] have used glass powder as an inhibitor for ASR, employing it as supplementary cementitious material. Compared to aggregate replacement, the replacement of cementitious materials with glass exerts a markedly different effect on the residual compressive strength of concrete after exposure to high temperatures. Belouadah et al. [17] argued that the pozzolanic reaction of glass powder transforms silicates into C-S-H, increasing the C-S-H content per unit volume of concrete; consequently, the decomposition of C-S-H at 500 °C renders waste glass concrete more sensitive to strength deterioration than ordinary concrete. However, excessive replacement of cement with glass powder significantly diminishes the initial strength of concrete, rendering high substitution rates unfeasible and thereby limiting the recycling potential of waste glass in construction materials.

In conclusion, when replacing a specific concrete material with glass alone (single-blended glass concrete), it often leads to bottleneck problems such as a sharp decrease in strength or occurrence ASR. Therefore, subsequent scholars have proposed waste glass double replacement of concrete, harnessing the pozzolanic activity of glass powder to mitigate the alkali–aggregate reaction induced by glass sand and thereby enhancing the efficiency of glass utilization. However, the use of a high content of glass inevitably affects the thermal properties and high-temperature resistance of concrete. In particular, the thermal conductivity of glass (0.93 W/m·K) is significantly lower than that of traditional granite aggregates (2.12~3.62 W/m·K) and natural sand (approximately 3.00 W/m·K) [18]. Flores-Ales et al. [19] suggested that the incorporation of waste glass delays the thermal transfer response of concrete, which can mitigate the thermal decomposition of the internal materials and reduce the internal damage of concrete. Lu et al. [20] argue that glass modified concrete exhibits no visible surface cracking when exposed to high temperatures of 800 °C, greatly alleviating the occurrence of spalling phenomena. In addition, when the temperature reaches above 550 °C, the glass particles gradually melt and soften, changing the concrete microstructure damage morphology. Li et al. [21] pointed out that the melting and reconsolidation process of the glass enhanced the microstructural morphology, resulting in a higher residual strength of glass cement pastes after high temperatures. However, the influence of glass on the residual strength of concrete after high temperatures varies depending on its mode of replacement, warranting further investigation into the high-temperature mechanical properties of concrete under different replacement strategies.

Obtaining reliable data on the thermal properties of concrete is the key to post-disaster restoration assessment, and the specific variation patterns of the thermal parameters of CGC with increasing temperature still require further investigation. In addition, the effect of the type of glass replacement on the mechanical properties of concrete after elevated temperatures has not been widely reported. This paper investigates the optimal substitution scheme by changing the replacement rates of glass sand and glass powder. The thermal properties of CGC and the residual strength of concrete after high temperatures under different substitution schemes were also investigated. This study focuses on evaluating features such as apparent characteristic, mass loss, residual compressive strength, pore structure distribution, and microstructure of waste glass concrete.

## 2. Material Characteristics and Experimental Program

### 2.1. Raw Materials

#### 2.1.1. Cementitious Materials

**Figure 1:**
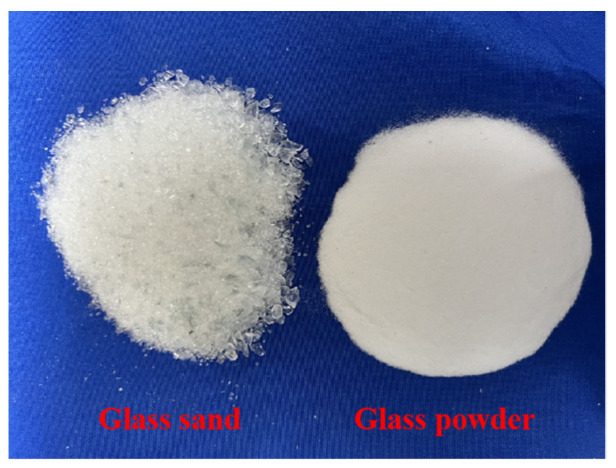
Physical appearance of glass sand and glass powder.

This study used P.O 42.5 ordinary Portland cement (produced by the Shaanxi Qinling Cement Co., Ltd., Tongchuan, China). The supplementary cementitious materials used in the experiment were derived from waste glass of Henan waste recycling station, which is made of sodium calcium glass. The glass was processed through impurity removal, crushing, ball milling, sieving, and drying. Glass powder with a particle size smaller than 75 μm was selected, as shown in Figure 1. The chemical composition of waste glass is shown in Table 1.

#### 2.1.2. Aggregates

**Figure 2:**
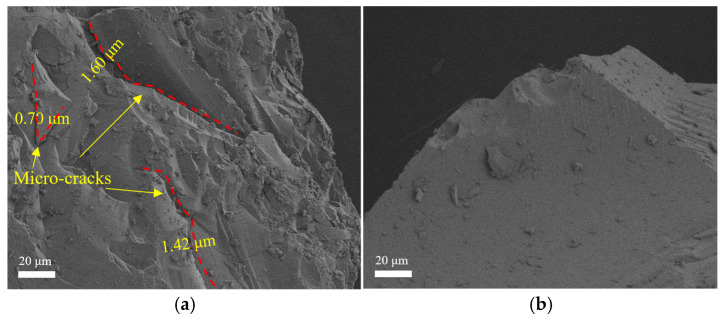
Microscopic characteristics of glass: (**a**) glass sand and (**b**) glass powder.

**Figure 3:**
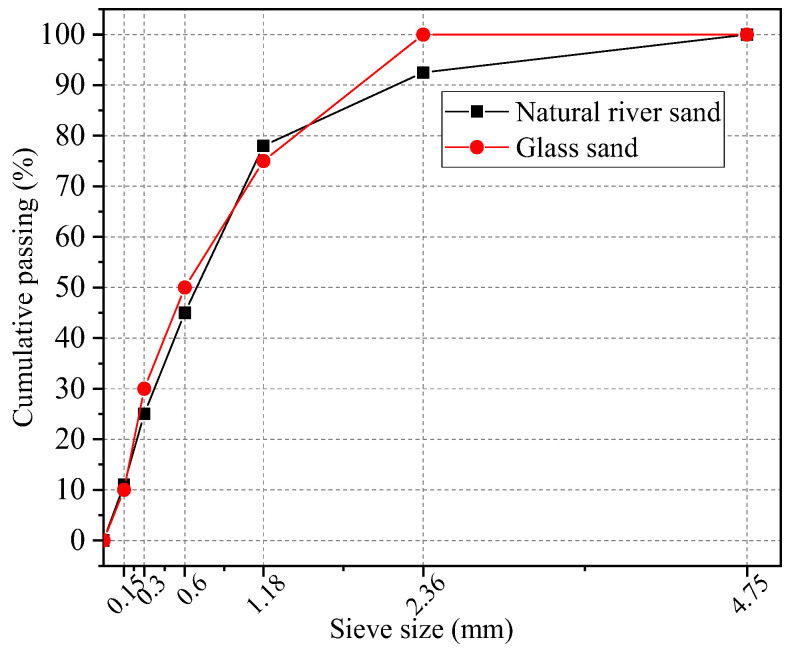
Grain size distribution of fine aggregate.

The experiment used natural gravel from Shaanxi with a continuous gradation of 5~20 mm as the coarse aggregate. Fine aggregates are made of natural river sand with a particle size less than 5 mm. Waste glass (made in Henan) was also used as a substitute for fine aggregates, ground and sieved according to the particle size distribution of Zone II medium sand. Microscopic observation revealed that large glass particles exhibited significant micro-cracks (Figure 2) [22]. To address these inherent defects, the glass sand particle size range was reduced to 0 to 2.36 mm. The particle size distribution curves of glass sand and natural river sand are shown in Figure 3. Additionally, Table 2 presents the physical properties of natural river sand and glass sand. The apparent density of glass sand is 2410 kg/m^3^, which is lower than the 2530 kg/m^3^ of natural river sand. Glass lacks water-absorbing groups, and the water absorption rate is only generated by capillary effect, which is much lower than the water absorption rate of natural river sand.

### 2.2. Concrete Mix Design

Three batches of concrete samples with a water–cement ratio of 0.5 were prepared, and a total of 15 mixing ratios were designed. For the first batch of samples, to ensure the maximum dosage of waste glass, the replacement rate of glass sand was prioritized and increased. Based on the research conclusions of some scholars on single-doped glass powder concrete (SPC), it is preliminarily determined that the glass powder content is 20% [2,23]. The replacement rate of glass sand instead of river sand was changed under the condition that glass powder replaced 20%. The density of glass sand is lower than that of natural river sand, so using equal mass replacement would cause an imbalance in aggregate proportions. Therefore, glass sand is replaced by volume replacement method of 0%, 10%, 20%, 30%, 40%, 50%, 60%, 70%, 80%, 90%, and 100% of fine aggregate, respectively.

For the second batch of samples, based on the first batch, determine the optimal proportion of glass powder. Vary the glass powder content at replacement rates of 0%, 10%, 20%, and 30% to ascertain the optimal amount of glass powder. The third batch of samples involves investigating the effect of different glass substitution methods on the mechanical properties of concrete after exposure to high temperatures. Table 3 shows the design of concrete mix proportions.

### 2.3. Specimen Preparation

The preparation steps for waste glass concrete are as follows: (i) Evenly blend glass sand and natural river sand, and also mix cement and glass powder evenly. (ii) Add coarse and fine aggregates into the mixer and stir for 30 s. (iii) Add half of the cementitious materials and half of the water, and continue mixing for another 30 s. (iv) Add the remaining cementitious materials and water, and mix for 3 min. Upon completion of mixing, pour the mixture into 150 × 150 × 150 mm cubic molds, and compact it using a vibrator. After being placed at ordinary temperature for 24 h, demold and place in a standard curing room for 28 days. Additionally, according to ASTM C1260 [24], prepare 25 × 25 × 280 mm mortar bars to test the ASR of waste glass concrete under optimal composite conditions.

### 2.4. Heating Method

**Figure 4:**
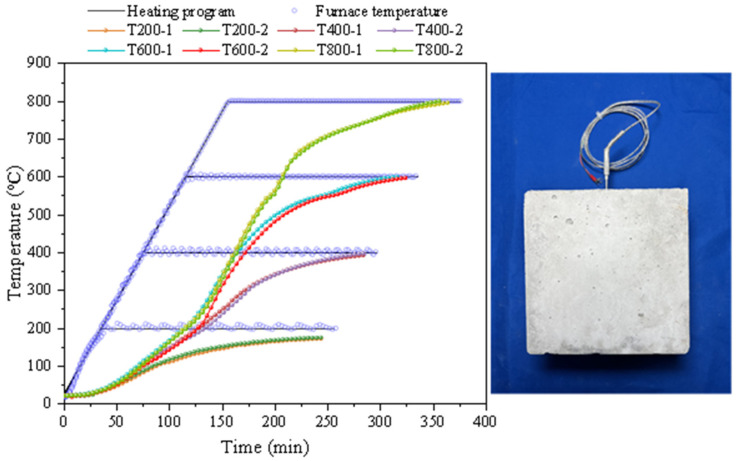
The heating-up curve of specimens.

To prevent excessive free water inside the concrete causing explosive spalling during high-temperature testing and affecting subsequent experiments, dry the specimens before conducting the tests. After drying the specimens in a 105 °C drying oven for 24 h, transfer them to a box-type resistance furnace for heating. The target temperatures are set to 200 °C, 400 °C, 600 °C, and 800 °C, with a reference temperature of ordinary temperature (25 °C). The rate of temperature increase in this test was 5 °C/min. To minimize internal temperature gradients within concrete, the constant temperature period was determined by temperature field tests. The thermocouple was embedded in the central region of the concrete specimen, and it was observed that the temperature at the center of the specimen lagged behind the furnace temperature. After 3.5 h of constant temperature, the core temperature of the specimen had essentially reached the target temperature, as shown in Figure 4. After heating, the specimen is naturally cooled to ordinary temperature along with the furnace.

### 2.5. Test Methods

#### 2.5.1. Mechanical Property Test

The mechanical performance is tested according to the current Chinese standard for physical and mechanical properties of concrete test methods GB/T 50081—2019 [25]. The experiment uses an electro-hydraulic pressure tester by Wuxi Xinluda Instrument Equipment Co., Ltd. (Wuxi, China) to load the specimens, and the loading rate for the compressive strength test is 0.5~0.6 MPa/s. The strength value of the specimens is the average of the results of the 3 specimens.

#### 2.5.2. Expansion Test

The rapid mortar rod method was used to test the ASR expansion rate. The specimens were cured in a 1 mol/L sodium hydroxide solution at (80 ± 2) °C according to standard requirements. The expansion rates (*ε*_t_) of each specimen at different ages (1 d, 3 d, 7 d, 10 d, 14 d) are measured, ensuring that the length measurement was completed within 15 s. The expansion rate is calculated according to Equation (1). If the expansion rate is less than 0.1% after 14 days, it is considered harmless.

(1) $$\varepsilon_{t}=\frac{L_{t}-L_{0}}{L_{0}-2\Delta }\times 100$$

where *L*_t_ is the length of the specimen at different ages; *L*_0_ is the initial length of the specimen; and *Δ* is the length of the probe.

#### 2.5.3. Mass Loss Test

Weigh the sample before and after the high-temperature test using an electronic scale with a precision of 0.1 g. Calculate the mass loss rate (*M*_lossT_) of the sample exposed to different temperatures using the following Equation (2):

(2) $$M_{lossT}=\frac{m_{0}-m_{cT}}{m_{0}}\times 100\%$$

where *m*_0_ is the quality of the sample at ordinary temperature, and *m*_cT_ is the quality of the sample after experiencing different high temperatures.

#### 2.5.4. Thermal Conductivity Test

**Figure 5:**
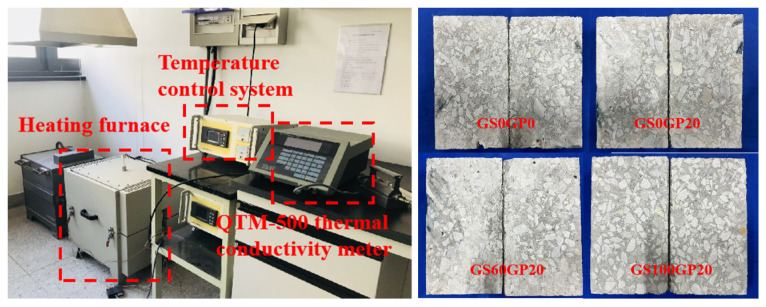
Thermal conductivity test.

The hot-wire method was used to measure the variation rule of thermal conductivity (*λ*) of CGC with temperature rise. The experimental equipment was obtained from QTM-500 hot-wire thermal conductivity meter of Shanghai Institute of Silicate, Chinese Academy of Sciences (Shanghai, China) (Figure 5), and the test process referred to GB/T 10297–2015 [26]. The hot-wire probe was clamped between two cubes with dimensions of 180 × 100 × 45 mm. The thermal conductivity was calculated based on the relationship between the temperature rise in the hot-wire and the time, as described by Equation (3). During the experiment, the furnace heating rate was controlled at 4 °C/min, with a temperature measurement range of 25~800 °C. Data were collected at intervals of 100 °C, and the thermal conductivity value was taken as the average of three measurements.

(3) $$\lambda =\frac{Q}{4\pi \left[T\left(t_{2}\right)-T\left(t_{1}\right)\right]}\ln\left(\frac{t_{2}}{t_{1}}\right)$$

where *Q* is the hot-wire power per unit length; *t*_1_ and *t*_2_ are the starting and ending time of hot-wire heating, respectively; and *T*(*t*_1_) and T(*t*_2_) are the hot-wire temperatures corresponding to time *t*_1_ and *t*_2_, respectively.

#### 2.5.5. Specific Heat Test

**Figure 6:**
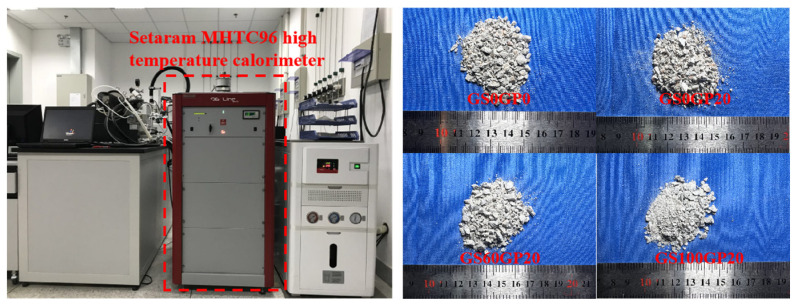
Specific heat test.

The specific heat (*C*_p_) of the concrete was measured using Differential Scanning Calorimetry (DSC). The test equipment was obtained from Setaram MHTC 96 high-temperature calorimeter of Shanghai Institute of Silicate, Chinese Academy of Sciences, and the test procedure was based on ASTM E1269-11 [27]. To reduce the randomness of the specimens, the aggregates and hydration products were ground into concrete fragments with diameters ranging from 0.5 to 2 mm, as shown in Figure 6. The furnace heating rate during the test was controlled at 10 °C/min, with a temperature measurement range of 180–800 °C. Data were collected at intervals of 20 °C, and the specific heat value was taken as the average of three sample measurements. The specific heat calculation formula is shown in Equation (4).

(4) $$C_{P}=C_{PC}\frac{m_{c}(A_{S}-A_{b})}{m_{s}(A_{c}-A_{b})}$$

where *C*_PC_ is the reference material’s specific heat, *m*_c_ is the reference material’s mass, *m*_s_ is the sample’s mass, *A*_c_ is the reference material’s heat flow, *A*_s_ is the sample’s heat flow, and *A*_b_ is the heat flow of the empty sample cell.

#### 2.5.6. Microstructure Observations

Utilize the Zeiss Sigma 300 scanning electron microscope (SEM) (Oberkochen, Germany) to observe the microstructure of the concrete. Select a small piece with a flat surface and a diameter of approximately 10 mm as the sample. Focus on observing the changes in the microstructure and interfacial transition zone (ITZ) of concrete exposed to different temperatures.

#### 2.5.7. Pore Structure Test

The pore structure of concrete was tested using a Macro MR12–150 H–I low-field nuclear magnetic resonance (NMR) instrument (Shanghai, China). Use the NMR phenomenon to detect the water signals in the water-filled pores of the sample to obtain the pore structure characteristics. To better study the changes in the internal pore structure of concrete after high temperatures, select cubes of approximately 10 mm × 10 mm × 10 mm from about 10 mm depth from the concrete surface as test samples. First, place the samples in a vacuum saturation machine for 24 h. Then, wipe the surface moisture of the sample to a dry state, and conduct the test in the NMR system.

## 3. Results and Analysis

### 3.1. Effect of Glass Materials on Concrete Performance

#### 3.1.1. Compressive Strength

**Figure 7:**
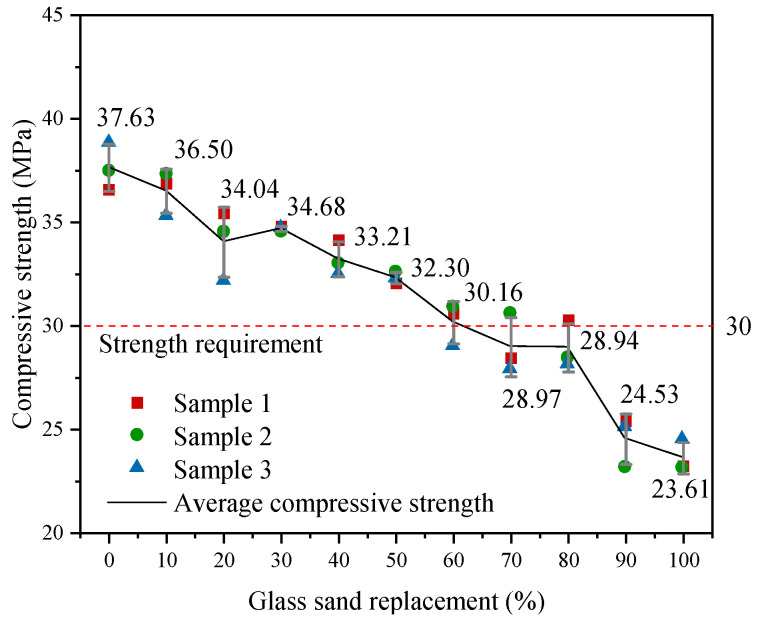
Compressive strength of samples with different glass sand content.

The variation in compressive strength of concrete with different glass sand replacement rates is shown in Figure 7. When the replacement rate increases, the compressive strength of the concrete decreases in a stepwise manner. Compared with 0% replacement rate, the compressive strengths of GS10GP20 and GS20GP20 drop significantly by 3.00% and 8.58%, respectively, indicating that the smooth surface of the glass weakens the adhesive force at the mortar–aggregate interface. Subsequently, as the replacement rate continues to increase, the rate of strength reduction slows down. This is related to the fact that the fineness modulus of glass sand is smaller than that of natural river sand, resulting in a denser concrete structure. When the waste glass content reaches 90%, the concrete strength sharply decreases. This is due to the low water absorption rate of glass sand, which increases the relative water–cement ratio. Under the influence of a high water–cement ratio and low apparent density, concrete experiences segregation and uneven aggregate distribution, leading to weak surface failure. To enhance the resource utilization rate of waste glass, a water–cement ratio of 0.5 for concrete must meet the 30 MPa standard value required by the Chinese standard GB 50010–2020 [28], thus selecting a glass sand content of 60%.

**Figure 8:**
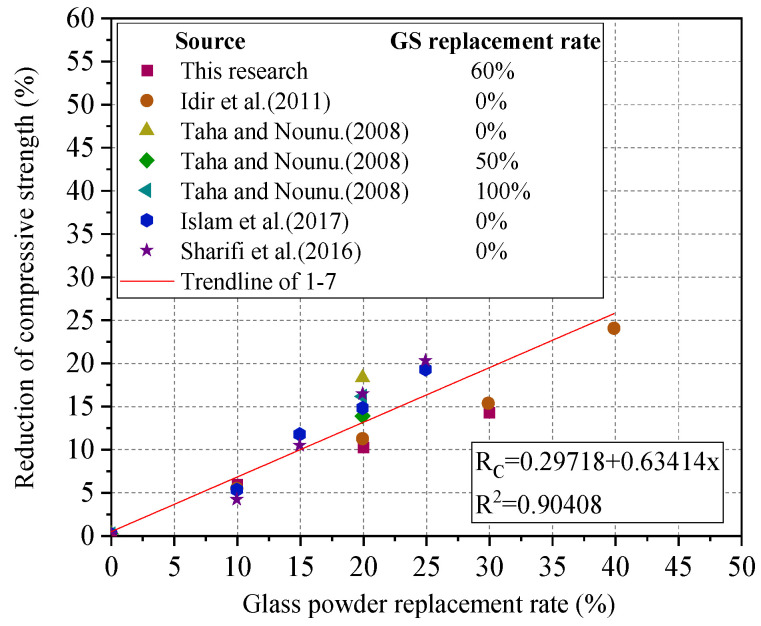
Compressive strength of samples with different glass powder content [2,29,30,31].

Based on the results of the first batch of samples, the replacement rate of glass powder was adjusted on the basis of 60% glass sand content to explore the variation in compressive strength. Existing research has shown a linear relationship between glass powder content and concrete strength [2,29,30,31]. Figure 8 summarizes the experimental results and gives the linear relationship equation. The reactivity of pozzolanic acid reaction was lower than that of cement hydration reaction at 28 d. Due to the dilution effect of replacing cement with glass powder, the 28-day strength of the concrete decreases linearly with the increase in replacement rate. When the glass powder content exceeds 20%, the strength drops below 30 MPa. Therefore, the optimal content for CGC is determined to be 60% glass sand and 20% glass powder. However, some researchers have concluded differently, suggesting that at low replacement rates of 10% glass powder, concrete exhibits higher compressive strength [32,33,34]. This discrepancy may be related to the glass particle size, with finer glass particles exhibiting higher pozzolanic activity and a more rapid pozzolanic reaction.

#### 3.1.2. Alkali–Silica Reactivity of Concrete

**Figure 9:**
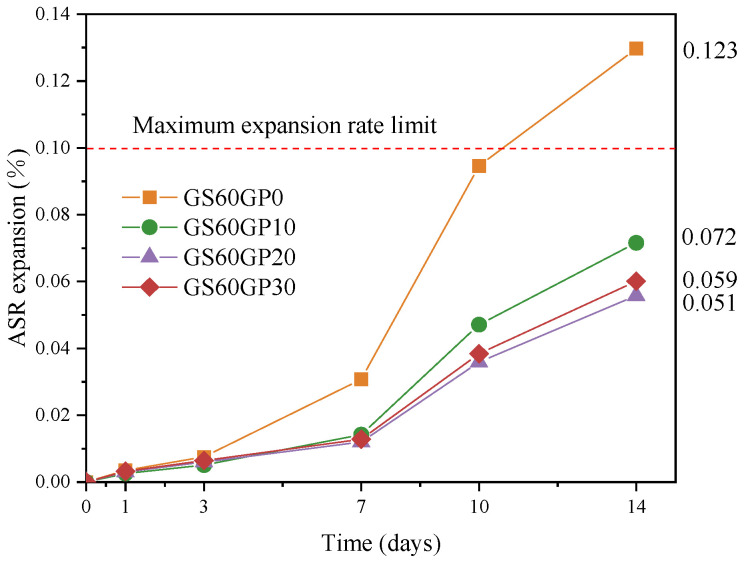
Expansion rate of specimens with different glass content.

The pozzolanic activity of glass powder consumes alkali bases, forming secondary C-S-H, which controls the available alkali content in the ASR [35], thereby improving the utilization rate of waste glass in concrete. To investigate the inhibitory effect of glass powder on the ASR, expansion tests were conducted on the second batch of samples. The test results are shown in Figure 9. It can be observed that the ASR expansion value of single-doped glass sand concrete (SSC) exceeds the normative threshold (0.1%) at 14 days [36]. The ASR expansion value of CGC is significantly reduced, indicating that glass powder has a significant inhibitory effect on ASR expansion. However, when the glass powder content exceeds 20%, the inhibitory effect on ASR expansion is no longer significant. Moreover, the results in Section 3.1.1 indicate that increasing the glass powder content causes a linear reduction in compressive strength. Therefore, the optimal content for CGC is determined to be 60% glass sand and 20% glass powder.

Research have shown that when glass alone replaces fine aggregate in concrete beyond 20%, the ASR becomes significant, and the durability is adversely affected [30]. The strength of single-doped glass powder concrete (SPC) decreases rapidly, severely limiting the utilization rate of waste glass [15,16]. The composite replacement method of waste glass not only solves the drawbacks of single replacement but also accounts for 17.79% of the mass of waste glass in concrete, significantly enhancing resource utilization. This approach can be used in structural components such as walls and slabs to consume a large amount of waste glass.

### 3.2. Thermal Properties

#### 3.2.1. Thermal Conductivity

**Figure 10:**
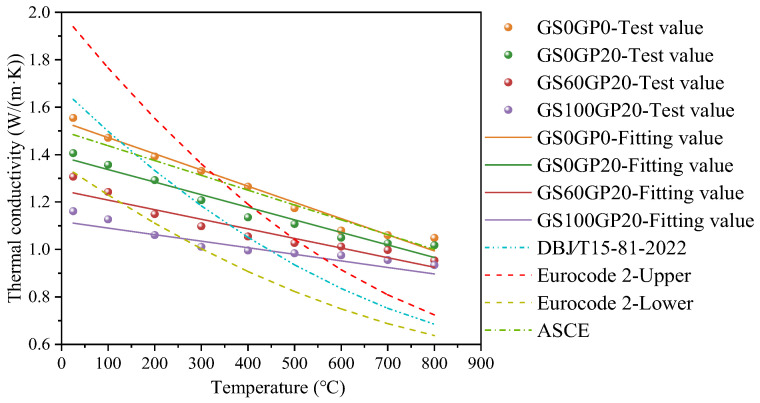
Relationship between thermal conductivity and temperature.

The type and content of concrete aggregates directly affect the thermal conductivity, thereby altering the heat transfer efficiency of the concrete. As shown in Figure 10, the thermal conductivity of concrete tends to decrease with the increase in waste glass admixture. This is because the thermal conductivity of glass (0.93 W/m·K) is lower than that of natural river sand (approximately 3.00 W/m·K). In addition, waste glass concrete has a relatively high porosity, which reduces the contact points within the matrix and thus lowers the heat transfer capacity of the concrete. With the temperature increases, the evaporation of free water and the decomposition of hydration products such as C-S-H and Ca(OH)_2_ lead to a reduction in the density of the concrete. In a sealed state, gases have a much lower thermal conductivity (0.023 W/m·K), which results in the thermal conductivity of the concrete decreasing linearly with increasing temperature. However, the incorporation of glass slows down the rate at which the thermal conductivity of the concrete decreases. This suggests that the insulating properties of the glass can help to reduce the rate of decomposition of hydration products, which in turn contributes to improving the high-temperature resistance of the concrete.

According to Figure 10, it can be observed that the thermal conductivity prediction models for concrete established based on different standards show certain differences. Among them, the variation pattern of the thermal conductivity of ordinary concrete in this experiment is basically the same as the prediction model of the American Standard ASCE No. 78 [37]. However, the prediction models from the following standards are less applicable to CGC. For instance, at ambient temperature, the thermal conductivity of GS100GP20 is only 75% of that of OC. Therefore, it is necessary to modify the prediction model for the thermal conductivity of CGC. Based on experimental data, it was found that the thermal conductivity of CGC is linearly related to the mass ratio of waste glass. Therefore, a prediction model for thermal conductivity that takes into account the mass ratio of glass and temperature has been proposed, which can be expressed by Equation (5).

(5) $$\lambda_{c}=1.48-1.30\omega +(12.72\omega -6.31)(T/10000)$$

where *λ*_c_ is the thermal conductivity of CGC; *ω* is the mass ratio of glass in concrete; and T is the temperature in Celsius.

#### 3.2.2. Specific Heat

**Figure 11:**
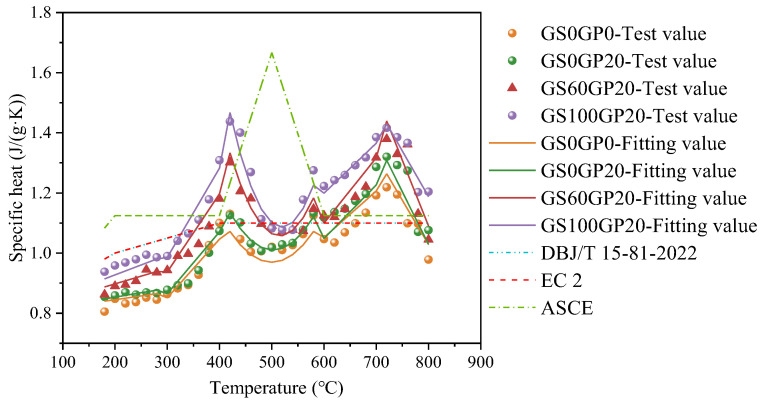
Relation between specific heat and temperature.

The specific heat capacity of concrete is closely related to water content, aggregate type, and density [38]. Figure 11 shows the variation curve of the specific heat capacity of waste glass concrete with temperature. It can be observed that the specific heat capacity increases fluctuatingly with rising temperature. The incorporation of glass increases the specific heat capacity of concrete. However, under different glass content levels, the variation pattern of the specific heat capacity with temperature remains largely consistent. Overall, the specific heat capacity of concrete exhibits three distinct peaks within the temperature range of 180–800 °C. The first peak occurs at 420 °C, which is attributed to the dehydration of calcium aluminate and C-S-H. The evaporation of chemically bound water in the concrete absorbs a large amount of heat. The second peak appears at 580 °C, at which point silica-based aggregates such as river sand and glass undergo a polymorphic transformation from quartz α to quartz β [39]. The third peak, observed at 720 °C, is caused by the decarbonation of CaCO_3_ in the aggregates, leading to a sharp change in the specific heat capacity of the concrete.

The high-temperature specific heat capacity of CGC shows significant differences when compared with prediction models from different regional standards. Specifically, Eurocode 2 [40] and DBJ/T 15–81–2022 [41] do not account for the quartz phase transition and the decarbonation of silicates due to differences in measurement techniques. These factors can lead to discrepancies in the predicted values of specific heat at high temperature. Based on experimental results, this paper establishes a prediction model for the specific heat capacity of concrete, which is related to the glass mass ratio and temperature. This model can accurately predict the variation of specific heat during the heating process, as represented by Equation (6).

(6) $$c_{c}=\left\{\begin{array}{c} 0.80+\left(2.50+14.78\omega \right)T/10000\\ 0.27-0.62\omega +\left(1.95+3.66\omega \right)T/1000\\ 4.98+23.26\omega -\left(1.60+8.59\omega \right)T/100+\left(1.61+8.02\omega \right)T^{2}/100000\\ 0.12-5.59\omega +21.08\omega^{2}+\left(1.55+9.00\omega -30.83\omega^{2}\right)T/1000\\ 3.38+17.22\omega -58.86\omega^{2}-\left(2.94-21.60\omega +76.38\omega^{2}\right)T/1000 \end{array}\right.\begin{array}{l} 180\;{}^{\circ}C\leq T\leq 200\;{}^{\circ}C\\ 200\;{}^{\circ}C<T\leq 400\;{}^{\circ}C\\ 400\;{}^{\circ}C<T\leq 580\;{}^{\circ}C\\ 580\;{}^{\circ}C<T<720\;{}^{\circ}C\\ 720\;{}^{\circ}C\leq T\leq 800\;{}^{\circ}C \end{array}$$

where *c*_c_ is the specific heat capacity of CGC; *ω* is the mass ratio of glass in concrete; and T is the temperature in Celsius.

### 3.3. High-Temperature Resistance

Based on the optimal substitution scheme, this study explores the effects of different substitution methods on the high-temperature performance of CGC. The focus is on evaluating key characteristics such as the apparent changes, mass loss, residual compressive strength, microstructure, and the distribution of pore structure in waste glass concrete.

#### 3.3.1. Apparent Characteristics

**Figure 12:**
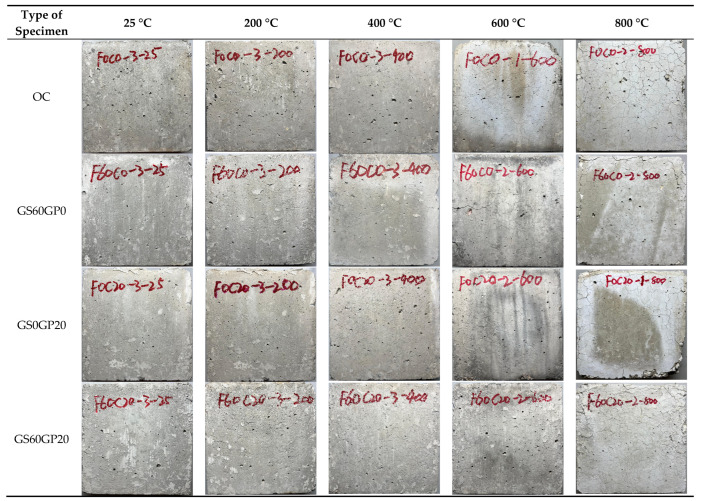
Apparent characteristics of concrete after high temperature.

High temperatures not only cause degradation of concrete materials but also increase internal stress, which can be manifested through the appearance characteristics of concrete. Therefore, the appearance characteristics of concrete after exposure to high temperatures can also reflect its high-temperature resistance performance, as shown in Figure 12. It can be seen from the figure that all types of concrete appear light gray at ordinary temperature. Compared to OC, waste glass concrete has a lighter color due to the milky white color of the glass material. When the temperature reaches 200 °C, the internal dehydration of the concrete causes the surface to darken to a deep gray. At this time, no visible cracks have appeared in the concrete, which is consistent with the research of other researchers [4]. As the temperature continues to rise to 400 °C, the concrete begins to turn yellow due to the oxidation of iron ions in the cement, forming iron oxides. At this temperature, small cracks begin to appear on the concrete surface, indicating that the concrete structure is damaged by high temperatures. With further temperature increases, the cracks gradually become apparent. At 600 °C, concrete shows significant cracking and surface spalling, with the surface turning light white. At 800 °C, the concrete has obvious falling blocks and missing corners, and the spalled cement is brittle into powder. The surface-exposed aggregate also appears milky white, due to the decomposition of CaCO_3_ in the aggregate. Overall, the changes in the appearance of various types of concrete after high-temperature exposure are basically consistent. The temperature dominates the apparent characteristics of concrete, while the replacement method has a minor impact.

#### 3.3.2. Mass Loss Rate

**Figure 13:**
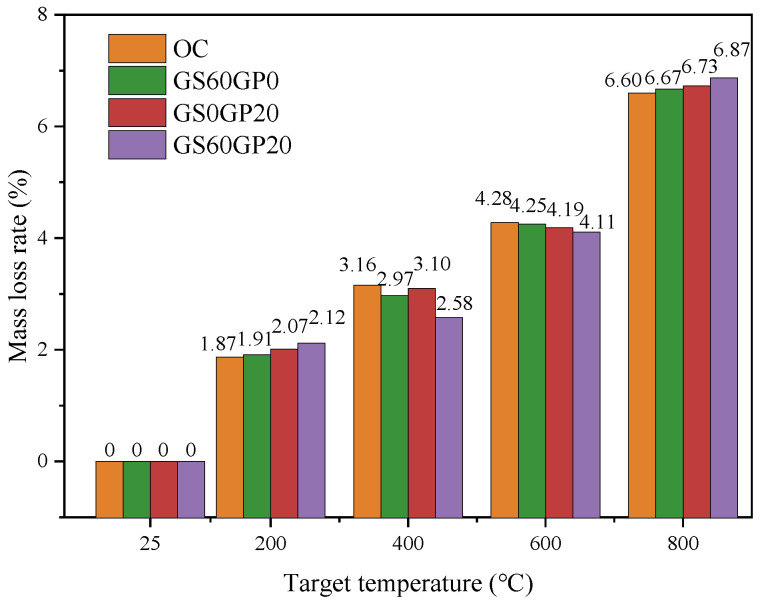
Mass loss rate of concrete after high temperature.

The relationship between the mass loss and temperature of waste glass concrete under different replacement methods is shown in Figure 13. The mass loss rates of various types of concrete increase with the increase in temperature. When concrete is heated to 200 °C, the mass loss is mainly caused by the evaporation of capillary water and the dehydration of ettringite [42]. The mass loss rates of concrete with different replacement methods are between 1.87% and 2.12%. Among them, the mass loss of SPC and CGC is slightly higher than SSC and OC, due to the non-absorbent characteristics of glass leading to more capillary water inside the concrete. Furthermore, the replacement of cement with glass powder produces a dilution effect, reducing the extent of the cement that reacts with water. As a result, the degree of cement hydration declines, leading to increased capillary water content within the concrete. When heated to 400 °C, the C-S-H begins to undergo chemical dehydration. As shown in Figure 13, SSC, SPC, and OC have similar mass loss rates, ranging from 2.97% to 3.16%. The CGC has a slightly lower mass loss rate at 2.41%. This is because CGC has a higher glass content and a lower thermal conductivity, slowing down the dehydration rate of C-S-H. In addition, glass has strong chemical stability and low degradation, which also improves the quality loss rate of the concrete.

During the temperature increase from 400 °C to 600 °C, Ca(OH)_2_ in the concrete decomposes, leading to a continued increase in mass loss rate. At 600 °C, the internal moisture within the concrete basically disappears [43], and the mass loss rates of various types of concrete tend to be consistent, ranging from 4.11% to 4.28%. When the temperature surpasses 600 °C, the C-S-H decomposes into dicalcium silicate, losing its cementing action, resulting in a sharp increase in the concrete mass loss rate [44]. In addition, CaCO_3_ in the aggregate decomposes into CaO and CO_2_ gas, which leads to an increase in the mass loss rate of concrete at 800 °C. At this temperature, the mass loss rate of waste glass concrete exceeds that of ordinary concrete. This is because the pozzolanic reaction of fine-grained glass powder is relatively low at 28 days, resulting in lower C-S-H content in waste glass concrete compared to OC. Therefore, the phenomenon of peeling caused by the decomposition of C-S-H becomes more pronounced [2].

#### 3.3.3. Failure Modes

**Figure 14:**
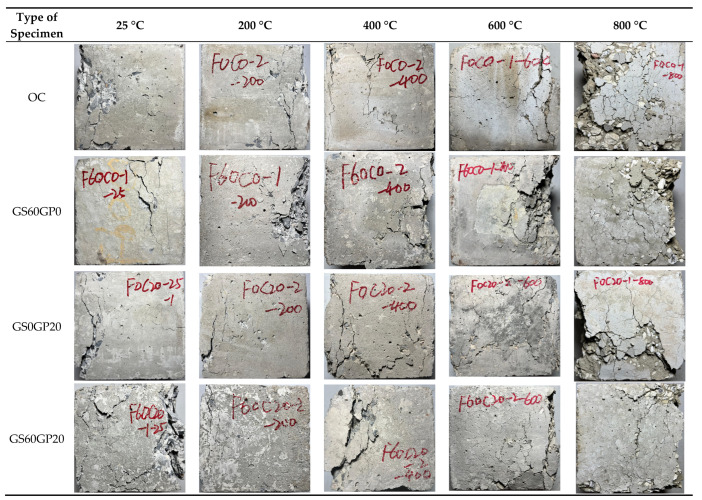
Failure mode of concrete after high temperature.

The compressive failure mode of concrete at different target temperatures is shown in Figure 14. The change trend of concrete failure modes with different replacement methods is consistent, and temperature effect is still the main factor affecting the failure mode of the concrete. When the temperature is below 600 °C, the concrete maintains good integrity, and the failure mode is along the main crack. When the temperature reaches above 600 °C, the decomposition of C-S-H leads to a significant decrease in the strength of the cement matrix, and the phenomenon of slip along cracks in concrete becomes more pronounced. In addition, concrete is accompanied by an increase in secondary cracks and severe spalling of fragments. As the temperature rises, the crack width between the aggregate and mortar also becomes larger. This is due to the more pronounced expansion of natural aggregates at higher temperatures.

#### 3.3.4. Residual Compressive Strength

**Figure 15:**
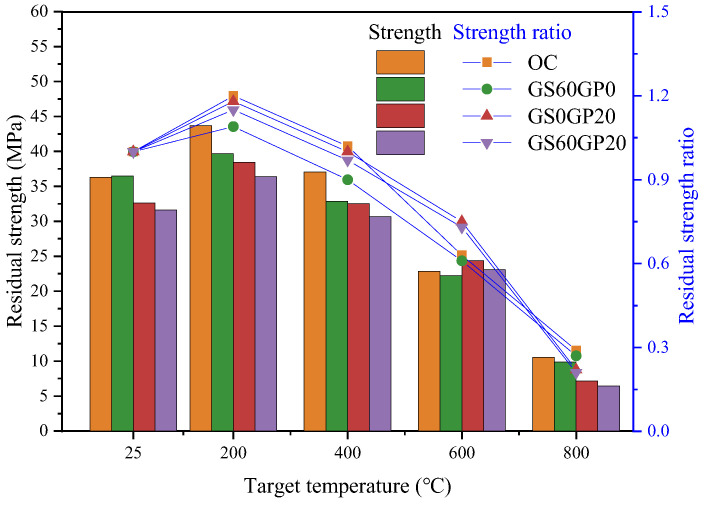
Variation in concrete strength after high temperature.

Concrete mainly bears compressive loads, and its residual compressive strength after disasters is crucial for evaluating its high-temperature resistance. Investigating the variations in compressive strength of concrete with different replacement methods of glass is advantageous for the practical application and promotion of waste glass concrete. The residual compressive strength of concrete and the strength reduction ratio are shown in Figure 15, where the strength reduction ratio is the ratio of compressive strength at the target temperature to that at ordinary temperature. Waste glass concrete exhibits a similar trend in strength variation to OC, showing a clear trend of first increase and then decrease [10].

At ordinary temperature, the compressive strengths of OC, GS60GP0, GS0GP20, and GS60GP20 are 36.29 MPa, 36.48 MPa, 32.60 MPa, and 31.62 MPa, respectively. Among them, the compressive strengths of GS0GP20 and GS60GP20 are slightly lower, owing to the pozzolanic reaction at 28 days exhibiting lower reactivity compared to the cement hydration reaction, which is consistent with the results observed under ambient temperature testing. When the temperature reaches 200 °C, the strengths of various types of concrete increase to varying degrees, attributed to high temperatures causing capillary water evaporation and form steam curing, which accelerates cement hydration reactions or the pozzolanic reaction of fine-grained glass powder. The residual strength ratio of SSC at 200 °C is only 1.09, which is significantly lower than that of OC. The capillary water evaporation increases concrete porosity, making the smooth surface characteristics of large glass particles more pronounced after high-temperature exposure. The pozzolanic reaction of fine-grained glass powder generates secondary C-S-H, improving the interface strength between concrete aggregates and mortar. Hence, there is a noticeable increase in the residual strength ratio of GS0GP20 and GS60GP20. After reaching 400 °C, the C-S-H begins to dehydrate and decompose, increasing internal porosity of the concrete and decreasing strength. At this time, the negative effects of concrete damage due to high temperatures gradually offset the advantages of steam curing. At 400 °C, the residual strength ratios of OC, GS60GP0, GS0GP20, and GS60GP20 are 1.02, 0.90, 1.00, and 0.97, respectively, indicating that the pozzolanic reaction in GS0GP20 and GS60GP20 can still improve the smooth surface characteristics of glass.

When the temperature rises to 600 °C, C-S-H and Ca(OH)_2_ continue to dehydrate and decompose, further reducing the strength of concrete. This is also reflected in the loosening of the internal structure of the concrete in the failure mode. At this time, the glass melts at high temperatures, acting as a binder to provide greater cohesion for concrete [45]. Among them, the OC strength reduction ratio decreases most significantly, which corresponds to the OC taking the lead in shifting to a lighter white color in the failure pattern. The residual strength ratios of GS0GP20 and GS60GP20 are slightly higher than that of GS60GP0, indicating that the melting effect of fine-grained glass powder is more significant under high-temperature conditions. When the temperature exceeds 600 °C, the decomposition of hydration products dominates the strength of concrete, and the C-S-H loses its cementing function after reaching 800 °C. Due to the lower reactivity of the pozzolanic reaction of fine-grained glass powder compared to hydration reactions at 28 days [2], the high-temperature decomposition of C-S-H has a more significant impact on compressive strength, resulting in a lower residual strength ratio of GS0GP20 (0.22) and GS60GP20 (0.21) compared to OC (0.29).

The above results indicate that the pozzolanic reaction in GS60GP20 can alleviate the smooth surface characteristics of glass sand under high temperatures. The melting effect of glass at 600 °C significantly improves the rate of strength decline in concrete. However, after 800 °C, the significant decomposition of C-S-H results in a decline rate in GS60GP20 exceeding that of OC.

#### 3.3.5. Microstructure Analysis

**Figure 16:**
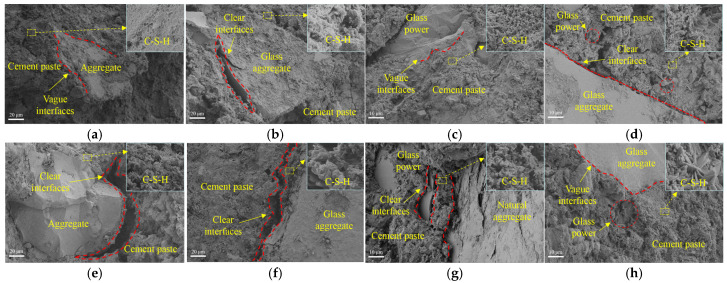
SEM images of concrete: (**a**) OC–600 °C, (**b**) GS60GP0–600 °C, (**c**) GS0GP20–600 °C, (**d**) GS60GP20–600 °C, (**e**) OC–800 °C, (**f**) GS60GP0–800 °C, (**g**) GS0GP20–800 °C, and (**h**) GS60GP20–800 °C.

Due to the significant influence of glass characteristics on the residual strength ratios of concrete at 600 °C and 800 °C, a comparative analysis of the concrete microstructure at these two temperatures is conducted, as shown in Figure 16. After exposure to 600 °C, compared to OC, the GS60GP0 exhibits more pronounced cracks at the interface between aggregates and cement paste. This is attributed to the smooth surface of glass aggregates and poor bonding with the cement paste, making the interface more susceptible to the effects of high-temperature decomposition of C-S-H, leading to interface cracking. In addition, the difference in thermal properties between glass aggregate and cement paste can also aggravate cracking. In GS0GP20, the fine-grained glass powder imparts pozzolanic activity due to the size effect, generating secondary C-S-H, thereby mitigating interface cracks between glass and the cement matrix. This macroscopically manifests as greater residual strength at high temperatures for GS0GP20. The larger glass aggregates in GS60GP20 still retain sharp edges, while the edges of fine-grained glass powder change with the shape of the pores. This indicates that there is a melting process of fine-grained glass powder at 600 °C, which heals the pores of the cement matrix. Therefore, fine-grained glass powder exhibits a stronger melting healing effect and can improve the residual strength ratio of concrete after 600 °C.

Figure 16e-h show the microstructural images of concrete under different replacement methods after exposure to 800 °C. The hydrated products transform from continuous flocculent to grainy with obvious voids, indicating that C-S-H is severely degraded under high temperatures. The clear cracks appear at the interface between aggregates and cement mortar in OC, which is related to the differences in thermal expansion between the cement mortar and aggregates. There are also obvious cracks at the interface of GS60GP0. However, compared to ordinary concrete, the surface of glass aggregates remains smooth without fine cracks or missing blocks, suggesting low degradation tendency of glass aggregates under high temperatures. The GS0GP20 produces obvious cracks at the interface and cement mortar, resulting in a sharp decrease in macroscopic mechanical properties. This indicates that the degree of decomposition of hydration products is the dominant factor in the strength of concrete at 800 °C. In GS60GP20, the edges of glass aggregates show no sharp angular features, indicating that the glass has undergone a melting and re-solidification process. However, this does not mitigate the decrease in concrete strength caused by the low content of hydrated products. Therefore, compared to OC, GS60GP20 requires a longer curing time to enhance the extent of the pozzolanic reaction.

#### 3.3.6. Pore Structure Analysis

**Figure 17:**
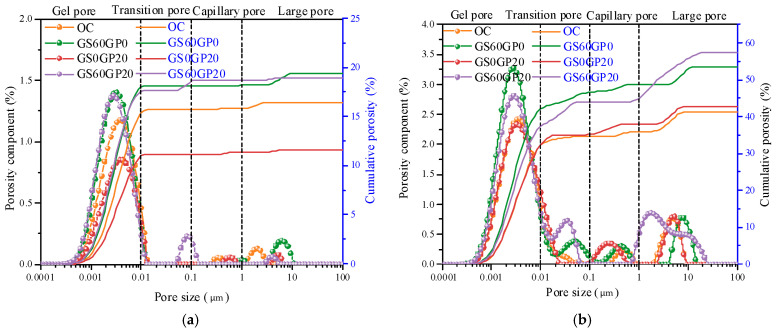
Effect of different replacement methods on pore structure of concrete at (**a**) 600 °C and (**b**) 800 °C.

The smooth surface and melting characteristics of glass affect the pore structure of the concrete matrix. Compared to OC, the pore structure of waste glass concrete is more complex, significantly impacting its mechanical properties [46]. Figure 17 shows the pore size distribution of concrete under different replacement methods after exposure to high temperatures. The pore sizes are categorized based on their impact on concrete performance: gel pores (<10 nm), transition pores (10–100 nm), capillary pores (100–1000 nm), and large pores (>1000 nm) [47]. At 600 °C, the cumulative porosity of GS60GP0 is significantly higher than that of OC, due to the smooth surface of the glass and its poor adhesion to the cement matrix, resulting in obvious interface cracks. Compared to OC, the cumulative porosity of GS0GP20 decreases, as the melting effect of fine glass powder plays a crucial role at this stage. Additionally, the melting effect of fine glass powder also reduces the cumulative porosity of GS60GP20 compared to GS60GP0. As C-S-H decomposes, the internal structure of the concrete becomes progressively looser, leading to the aggregation of pores into harmful pores (>50 nm) [48]. Therefore, at 800 °C, the cumulative porosity of all types of concrete increases, and the cumulative porosity of concrete under different replacement methods is proportional to the glass content.

**Figure 18:**
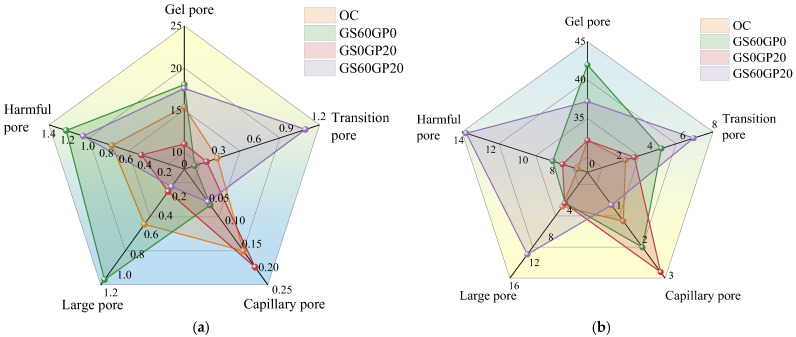
Comparison of concrete pore sizes under different replacement methods at (**a**) 600 °C and (**b**) 800 °C.

Figure 18 compares the various pore sizes of concrete under different replacement methods. After exposure to 600 °C, the number of large pores in GS60GP0 is significantly higher than in OC. This result also explains the low residual compressive strength of GS60GP0 after exposure to 600 °C. The gel pores are mainly composed of gel pores and inter-gel pores, and the inert glass powder reduces the degree of hydration in the concrete, thereby increasing the number of gel pores in GS60GP20. The melting effect of fine-glass powder occurs at 600 °C, causing the transformation of large pores into capillary pores, indicating a gradual reduction in concrete pore size. This also leads to higher residual strength ratios for GS0GP20 and GS60GP20 at 600 °C. Furthermore, fine-glass powder can reduce the number of harmful pores in the concrete, with the harmful pore porosity of GS60GP20 being 1.04%, which is only 85.5% of that in GS60GP0.

After exposure to 800 °C, the hydration products and aggregates continue to decompose, and the number of various pore sizes in concrete significantly increases. Compared to OC, GS60GP0 exhibits a significant increase in gel, transition, and capillary pores. In GS0GP20 and GS60GP20, due to the lower quantity of hydration products, gel pores gradually aggregate into large pores at high temperatures, resulting in an increase in harmful pores and greater damage. Therefore, after reaching a temperature of 800 °C, the degree of decomposition of hydration products determines the mechanical properties of the concrete, and the melting effect of glass cannot significantly improve concrete strength. At this point, the harmful pore porosity of GS60GP20 reaches its peak at 13.73%.

## 4. Conclusions

This study aims to prepare CGC by glass sand and glass powder. This study explored the variation in thermal conductivity and specific heat capacity of CGC with temperature, and the high-temperature resistance performance of the concrete under different replacement methods was investigated. The main conclusions of this paper are as follows:

1. The pozzolanic reaction of glass powder significantly relieves the ASR expansion rate in waste glass concrete, which solves the drawbacks of single-blended glass concrete. The glass content can reach 17.79% of the total concrete mass.
2. The thermal properties of CGC are closely related to the type of aggregate and the proportion of glass incorporated. Specifically, the thermal conductivity of CGC exhibits a linear correlation with temperature. The specific heat capacity shows three distinct peaks within the temperature range of 180 °C to 800 °C, which are attributed to chemical dehydration, quartz phase transitions, and the decarbonation of CaCO_3_, respectively.
3. The temperature plays a predominant role in influencing the apparent characteristics and failure modes of concrete, while the impact of replacement methods is relatively minor. As the temperature increases, the concrete gradually transitions from light gray to dark gray, gray-yellow, and finally light white. When the temperature reaches above 600 °C, the decomposition of C-S-H in the concrete leads to the formation of pronounced cracks and spalling.
4. The residual compressive strength ratios of CGC have a significant advantage at 600 °C (0.73), as the fine glass powder begins to melt and soften at this stage, enhancing its function as a binder. When the temperature reaches 800 °C, the cement matrix becomes brittle, and the decomposition of hydration products dominates concrete strength. At this time, the CGC residual intensity ratio is lower than that of OC.
5. The smooth surface of large-size glass aggregates makes the aggregate interface more susceptible to high-temperature decomposition of calcium silicate, leading to interface cracks. The fine-grained glass powder exhibits stronger melting effects, significantly improving the residual strength ratio of concrete after exposure to 600 °C. At 800 °C, the edges of glass aggregates no longer have obvious angular features. However, the melting effect of glass cannot alleviate the decrease in concrete strength caused by low hydration product content.
6. The inert glass powder reduces the degree of hydration in the concrete, thereby increasing the number of gel pores in CGC. However, the melting effect of glass can reduce concrete pore size, transforming large pores into capillary pores.

## Figures and Tables

**Table 1:** Chemical composition of waste glass.

Material	Constituents (%)							
	SiO_2_	CaO	Al_2_O_3_	Fe_2_O_3_	MgO	SO_3_	Na_2_O	K_2_O
Glass	72.86	11.58	1.84	0.84	1.92	0.34	10.10	0.45

**Table 2:** The physical characteristics of fine aggregate.

Material	Apparent Density (kg/m^3^)	Fineness Modulus	Water Absorption (%)
Natural river sand	2530	2.55	2.01
Glass sand	2410	2.35	0.32

**Table 3:** Mix proportions of waste glass concrete (kg/m^3^).

Type of Concrete	Water	Cement	Glass Powder	Sand	Glass Sand	Coarse Aggregate
OC	175	343	0	621	0	1261
GS0GP20	175	274.4	68.6	621	0	1261
GS10GP20	175	274.4	68.6	558.9	59.2	1261
GS20GP20	175	274.4	68.6	496.8	118.4	1261
GS30GP20	175	274.4	68.6	434.7	177.6	1261
GS40GP20	175	274.4	68.6	372.6	236.8	1261
GS50GP20	175	274.4	68.6	310.5	296	1261
GS60GP20	175	274.4	68.6	248.4	355.2	1261
GS70GP20	175	274.4	68.6	186.3	414.4	1261
GS80GP20	175	274.4	68.6	124.2	473.6	1261
GS90GP20	175	274.4	68.6	62.1	532.8	1261
GS100GP20	175	274.4	68.6	0	592	1261
GS60GP0	175	343	0	248.4	355.2	1261
GS60GP10	175	308.7	34.3	248.4	355.2	1261
GS60GP30	175	240.1	102.9	248.4	355.2	1261

Notes: The second and third batches are determined by subsequent experimental results; GS: glass sand; GP: glass powder.

## Data Availability

The raw data supporting the conclusions of this article will be made available by the authors on request.
